# Emerging horizons in cancer therapy: Squamous transition drives drug resistance

**DOI:** 10.1002/ctm2.1697

**Published:** 2024-06-22

**Authors:** Ningxia Zhang, Xinyuan Tong, Hongbin Ji

**Affiliations:** ^1^ Department of Respiratory and Critical Care Medicine The Fourth Affiliated Hospital, Zhejiang University School of Medicine Yiwu China; ^2^ Key Laboratory of Multi‐Cell Systems, Shanghai Institute of Biochemistry and Cell Biology Center for Excellence in Molecular Cell Science, Chinese Academy of Sciences Shanghai China; ^3^ School of Life Science, Hangzhou Institute for Advanced Study University of Chinese Academy of Sciences Hangzhou China; ^4^ School of Life Science and Technology ShanghaiTech University Shanghai China

**Keywords:** Adeno‐to‐squamous transition (AST), Drug Resistance, Targeted Therapy

## Abstract

Adeno‐to‐squamous transition (AST) has emerged as a driver of targeted therapy resistance. Studies of KRAS/LKB1‐mutant non‐small cell lung cancer (NSCLC) have revealed the progressive acquisition of squamous signatures during treatment with KRAS inhibitors, ultimately inducing drug resistance. Moreover, the expression of squamous‐related gene KRT6A in adenocarcinoma (ADC) correlates with poor responses to KRAS inhibitors. In this commentary, we discuss the role of AST in drug resistance and propose future directions to elucidate the underlying mechanisms.

**Key points:**

- Adeno‐to‐squamous transition (AST) drives targeted therapy resistance.
- Progressive plasticity is acquired during Adeno‐to‐squamous transition (AST).

## 1 THE CHALLENGE OF TARGETED THERAPY

With the identification of more and more oncogenic driver genes in lung cancer, targeted therapies have revolutionized the approach to treating tumours. These therapies are highly selective and effective with fewer adverse effects and offer significant advantages over traditional chemotherapy. However, tumour cells may still evolve over time, employing mechanisms like secondary mutations or alternative signalling pathways, which undermine these targeted therapies and lead to disease progression. This highlights an urgent need for innovative approaches to overcome drug resistance.

In epidermal growth factor receptor (EGFR)‐mutant non‐small cell lung cancer (NSCLC), resistance to first‐ and second‐generation EGFR tyrosine kinase inhibitors (TKIs) often occurs due to the T790M mutation in its kinase domain. Notably, the phenotypic transition has emerged as an alternative resistance mechanism in a clinic, including the epithelial‐to‐mesenchymal transition (EMT), the transformation from NSCLC to SCLC, and the adeno‐to‐squamous transition (AST). These observations underscore the importance of lineage plasticity in orchestrating targeted therapy resistance although the precise mechanisms driving these transitions remain largely unexplored.

## 2 AST DRIVES TARGETED THERAPY RESISTENCE

Recent studies have highlighted a connection between AST and the resistance to KRAS^G12C^ targeted therapy. In the phase 1/2 KRYSTAL‐1 clinical trial, which tested the KRAS^G12C^ inhibitor Adagrasib on NSCLC patients, two out of nine lung adenocarcinoma (ADC) patients displayed squamous pathology upon re‐biopsies after relapse.^1^ We analyze the transcriptome data of pre‐treatment biopsies from 68 patients and identify an inverse relationship between the expression of a squamous cell carcinoma (SCC) gene signature and the duration of Adagrasib treatment. Strikingly, this correlation is only detectable in the subgroup of patients with concurrent mutations of *STK11*/*LKB1*. Using *KRAS^G12C^* and *Kras^G12D^* genetically engineered mouse models as well as organoid models, we further find that ΔNp63, the mast transcription factor (TF) of SCC, is able to drive the squamous transition and the resistance to KRAS inhibitors, whereas the ETS‐related transcription factor ELF5 plays an important role in maintaining the ADC lineage and the sensitivity to KRAS‐mutant targeted therapy.^2^

Squamous transition has also been observed in patients with other oncogenic drivers such as EGFR mutations and EML4‐ALK fusions. Studies on EGFR‐mutant lung cancer show that squamous transition is an arising resistance mechanism of first‐line and late‐line Osimertinib therapies, in addition to other molecular mechanisms including PIK3CA mutations, chromosome 3q amplification, and FGF amplification.^3^ Moreover, the activation of JAK‐STAT signalling in EML4‐ALK fusion‐driven tumours has also been linked to promoting squamous transition, which contributes to resistance to targeted therapies.^4^ These findings highlight that AST is a drug resistance mechanism independent of the variety of oncogenic drivers, and understanding and addressing such lineage plasticity could provide novel approaches to overcome AST‐mediated drug resistance.

## 3 PROGRESSIVE PLASTICITY DURING RESISTENCE

**FIGURE 1:**
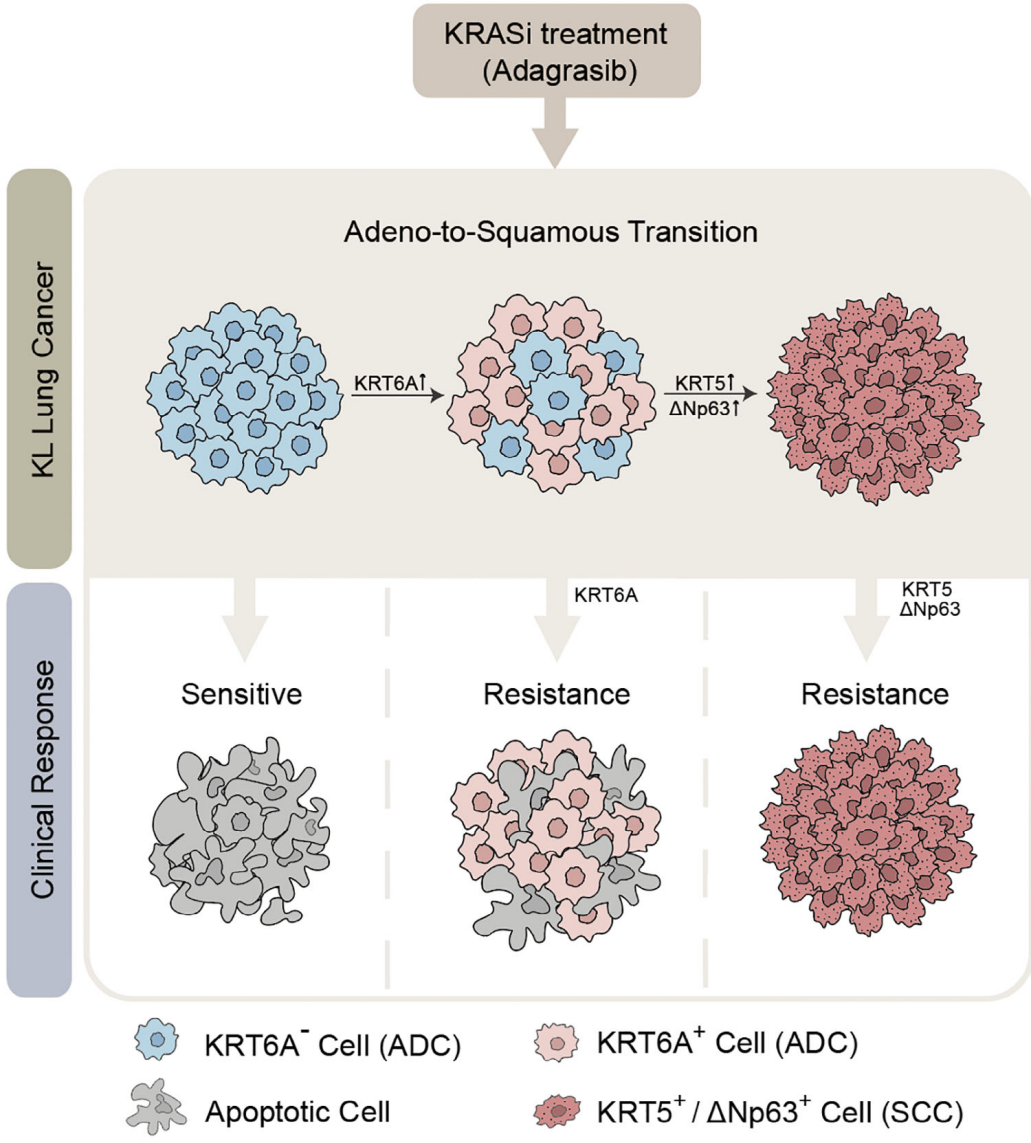
AST drives KRAS inhibitor resistance in KRAS/LKB1‐mutant lung cancer. Lung ADC, featured with negative KRT6A expression, are initially sensitive to KRAS inhibitor Adagrasib and undergo dramatic apoptosis after targeted therapy. With continuous Adagrasib treatments, certain lung ADC cells might up‐regulate the expression of KRT6A, entering a plastic cancer state while the tumour still pathologically resembles adenocarcinoma. Once fully transitioning into SCC, the tumour acquires the expression of KRT5 as well as the squamous lineage transcription factor ΔNp63 and develops strong resistance to Adagrasib. KL, KRAS/LKB1 mutant; ADC, adenocarcinoma; SCC, squamous cell carcinoma.

In our studies using plastic organoids derived from K_D_L mice (*Kras^LSL‐G12D/+^; Lkb1^flox/flox^*), we observed a progressive increase in the expression of SCC markers ΔNp63 and KRT5 over successive passages and a progressive decrease of the expression of ADC TF NKX2‐1,^2^ consistent with our previous findings in human clinical samples and mouse models.^5^, ^6^, ^7^, ^8^ These findings indicate that AST‐mediated resistance evolves gradually in the organoid system, potentially providing an opportunity to identify specific markers preceding squamous transition. Notably, we discover that KRT6A expression is significantly elevated in transitioning organoids, which precedes the full development of squamous tumours with the expression of ΔNp63 and KRT5 as well as the drug resistance (Figure 1). Moreover, the upregulation of KRT6A is observed in the tumours pathologically resembling ADC. Importantly, the KRT6A expression is inversely related to Adagrasib responses in the KRYSTAL‐1 clinical trial. Our data show that KRT6A could serve as an early predictive marker for AST and resistance to KRAS inhibitors.^2^ Although future study of a larger cohort is necessary to confirm our findings, we believe that the identification of KRT6A as a biomarker for timing prediction of drug response will help the development of novel strategies in managing these KRAS‐mutant lung cancer patients.

Our data demonstrate that KRT6A expression in ADC predicts the potential evolution path towards AST and drug resistance, which is much earlier than the appearance of the squamous markers ΔNp63 and KRT5 in fully developed SCC. Interestingly, such a phenomenon seems not exclusive to lung cancer. For instance, a transition to a semi‐squamous state was recently observed during the chemoresistance acquisition of muscle‐invasive bladder cancer and these resistant tumours display the expression of squamous markers ΔNp63, KRT5 and KRT14.^9^ Moreover, the non‐canonical metastases of colorectal cancer (CRC) are found to undergo squamous transition with the appearance of KRT5 expression without notable ΔNp63 upregulation.^10^

## 4 FUTURE OUTLOOK

Accumulating evidence indicates that lineage plasticity goes beyond lung cancer. It appears to be a universal biological process across various cancers including bladder cancer and CRC. Such findings offer a new horizon through which we might uncover new cancer biology, heterogeneity and plasticity. Future efforts will be necessary to uncover the underlying mechanisms driving such phenotypic transition. Previous studies have mainly focused on cancer cell‐intrinsic signalling, overlooking the potential influence of the tumour microenvironment. Given the significant modification to the microenvironment under therapeutic pressure, future exploration of the impacts of various types of non‐cancer cells, for example, immune cells and cancer‐associated fibroblasts, represents a compelling and important direction to understand the AST process.

## AUTHOR CONTRIBUTIONS

Ningxia Zhang and Hongbin Ji conceived the idea and wrote the manuscript. Xinyuan Tong and Hongbin Ji revised the manuscript. All authors read and approved the final manuscript.

## CONFLICT OF INTEREST STATEMENT

The authors declare no conflict of interest.

## ETHICS STATEMENT

Not applicable.
